# Pelvic MRI and CT images are interchangeable for measuring peripouch fat

**DOI:** 10.1038/s41598-017-12732-6

**Published:** 2017-09-29

**Authors:** Xian Hua Gao, Nan Lan, Hanumant Chouhan, Luca Stocchi, Erick Remer, Bo Shen

**Affiliations:** 1 Department of Colorectal Surgery, Changhai Hospital, Shanghai, China; 2 Department of Colorectal Surgery, the Cleveland Clinic Foundation, Cleveland, OH USA; 3 Department of Gastroenterology/Hepatology, the Cleveland Clinic Foundation, Cleveland, OH USA; 4 Department of Abdominal Imaging, the Cleveland Clinic Foundation, Cleveland, OH USA

## Abstract

A total of 27 pouch patients with inflammatory bowel diseases, who underwent pelvic MRI-DIXON and CT scan within one year, were included. Peripouch fat areas were measured at the middle height level of pouch (AreaM) and the highest level of pouch (AreaH). Our results demonstrated that measurements of perianal fat thickness, AreaM and AreaH based on MRI image were accurate and reproducible (correlation efficiency(r): intraobserver: 0.984–0.991; interobserver: 0.969–0.971; all P < 0.001). Bland-Altman analysis showed that more than 92.593% (25/27) of dots fell within the limits of agreement. We also identified strong agreements between CT and MRI image in measuring perianal fat thickness(r = 0.823, P < 0.001), AreaM (r = 0.773, P < 0.001) and AreaH (r = 0.862, P < 0.001). Interchangeable calculating formula to normalize measurements between CT and MRI images were created: Thickness_CT = 0.610 × Thickness_MRI + 0.853; AreaM_CT = 0.865 × AreaM_MRI + 1.392; AreaH_CT = 0.508 × AreaH_MRI + 15.001. In conclusion, pelvic MRI image is a feasible and reproducible method for quantifying peripouch fat. Pelvic MRI and CT images are interchangeable in retrospective measurements of peripouch fat, which will foster future investigation of the role of mesentery fat in colorectal diseases.

## Introduction

Ulcerative colitis (UC) is a lifelong disease arising from an abnormal interaction between genetic, environmental, and immunological factors^1^. There is a tendency of increased incidence in recent years^2^. The advances in medical therapy appear to alter the natural history of UC, leading to a decreased trend of colectomy^3,4^. However, for patients with refractory UC and UC with neoplasia, colectomy is inevitable. It is estimated that colectomy would be ultimately required in approximately 20% of UC patients^5^. Total proctocolectomy with ileal pouch-anal anastomosis (IPAA) is the gold standard surgical procedure for UC^4–7^. Although IPAA improves patients’ quality of life, pouch-related complications can occur, including chronic pouchitis, Crohn’s disease (CD) of pouch and pouch fistula. Those pouch-related complications may result in pouch failure, requiring pouch excision, pouch revision or permanent stoma^7^.

Obesity and abdominal visceral fat have been shown to contribute to chronic pouchitis, pouch anastomotic sinus, and pouch failure^8–11^. On the other hand, the impact of mesenteric fat on disease course of inflammatory bowel disease (IBD) has attracted an increasing attention^12–14^. Mesentery fat plays an active role in immune responses of intestinal inflammation and in host’s defenses against intestinal bacterial translocation^12,15–17^. Peripouch visceral fat, as a major composition of mesentery, was shown to be associated with pouch complication and pouch failure in our recent study based on MRI imaging (submission under review).

Accurate quantification of peripouch fat using existed CT/MRI image is desirable because it could provide us much valuable information without additional cost. Both CT and MRI have been reported in measuring abdominal visceral fat with high accuracy and reliability^9,10,18–24^. Moreover, measurements of abdominal visceral fat calculated from CT and MRI have been shown to have a close correlation based on pre-defined research protocol^18,20,25–27^. However, no study has reported measuring peripouch fat based on retrospective review of pelvic CT images. Furthermore, it is unclear whether CT and MRI image modalities are interchangeable in measuring peripouch fat. In our previous study (manuscript under review), we used pelvic MRI-DIXON-F image to measure peripouch fat area for the first time. The aims of this study were to validate retrospective measuring of peripouch fat with pelvic MRI-DIXON-F images, and to explore interchangeability between pelvic MRI and CT imaging for measuring peripouch fat.

## Results

**Figure 1:**
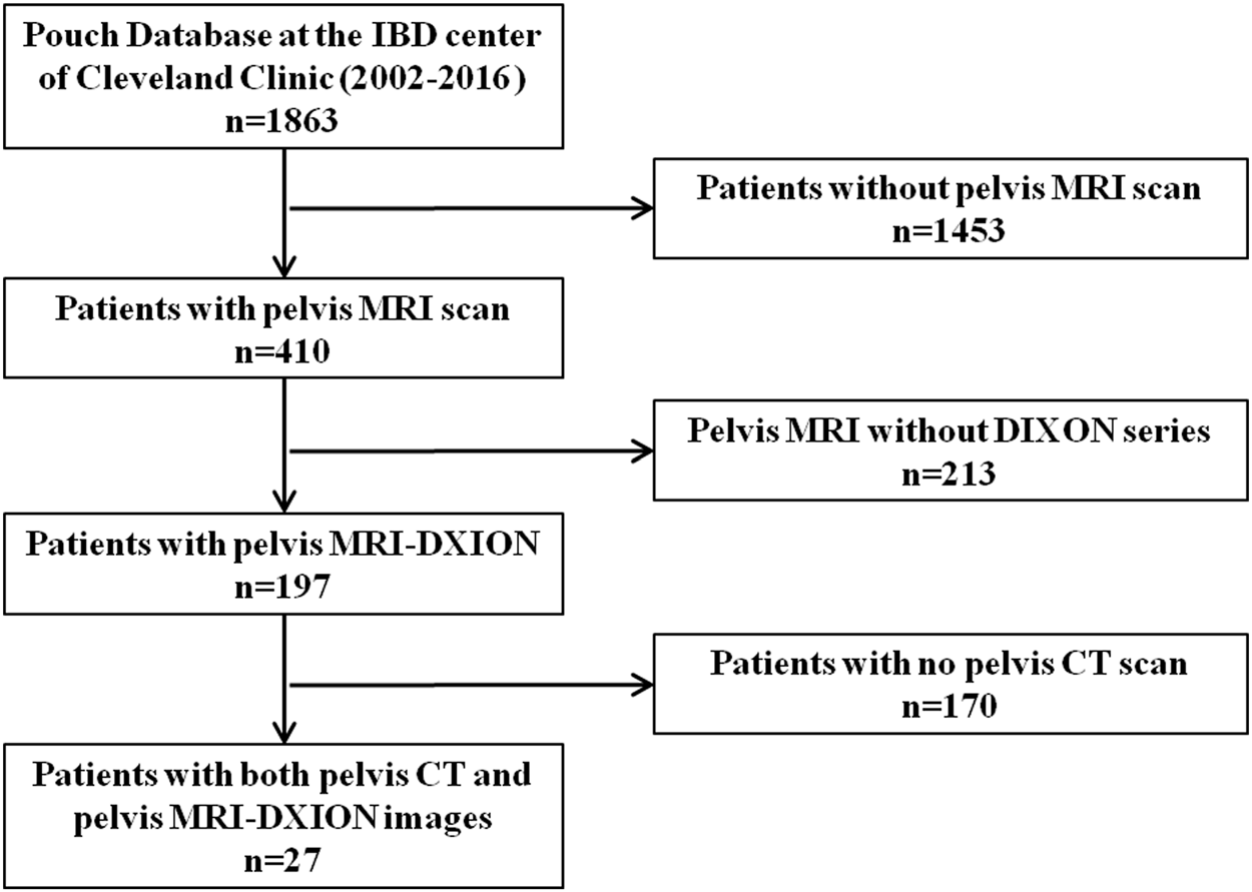
Flow chart of patient selection.

A total of 27 patients who had both pelvic MRI-DIXON-F and CT were included. The flow chart of patient selection was showed in Fig. 1. Briefly, of the 1,863 IBD patients in our pouch database, 410 had pelvic MRI, 197 had pelvic MRI-DIXON-F. Of the 197 patients, only 27 had undergone both pelvic CT scan and pelvic MRI-DIXON-F scan within one year, and all of these 27 patients were included. A total of 162 images (27 patients × 3 levels × 2 scans) were analyzed. These images were performed between April, 2009 and October, 2015. Of the included 27 patients, 14 were female and 13 were male; 26(96.3%) were J pouch. The median time interval between pelvic CT scans and pelvic MRI-DIXON-F scans was 20 days (interquartile range (IQR): −90 to 70 days). The median age at IBD diagnosis was 21(14–35) years; the median age at pouch construction was 36(26–47) years. The detailed demographic and characteristics of the 27 included patients were shown in Table 1. There were no significant differences between the two groups in terms of age at imaging (41(35–57)vs. 42(36–57) y, P = 0.978), time from pouch construction to imaging (4(1–10)vs. 4(1–10) y, P = 0.986), weight at imaging (69.501 ± 16.698vs. 68.576 ± 16.297 Kg, P = 0.838), height at imaging (1.694 ± 0.113 vs. 1.691 ± 0.114 m, P = 0.899) and body mass index (BMI) at imaging (24.155 ± 5.323vs. 23.890 ± 4.754 Kg/m^2^,P = 0.848) (Table 2).

**Table 1:** The demographic and characteristics of the 27 included patients.

Parameters		Values	Parameters		Values
Age at IBD diagnosis (y)		21(14–35)	Preoperative biologics	No	14(51.9%)
Age at pouch construction (y)		36(26–47)		Yes	13(48.1%)
Time from diagnosis to pouch construction (y)		9 (4–20)	Postoperative immunomodulator	No	25(92.6%)
Pouch survival (m)		48 (24–96)		Yes	2(7.4%)
Time from CT imaging to MRI imaging (d)		20(−90–70)	Postoperative biologics	No	24(88.9%)
Pouch failure	No	13(48.1%)		Yes	3(11.1%)
	Yes	14(51.9%)	Extensive colitis	No	0(0%)
Final diagnosis	Normal pouch	3(11.1%)		Yes	27(100%)
	IPS	5(18.5%)	Toxic megacolon	No	26(96.3%)
	Acute pouchitis	1(3.7%)		Yes	1(3.7%)
	Refractory pouchitis	4(14.8%)	Pouch type	J pouch	26(96.3%)
	Cuffits	5(18.5%)		others	1(3.7%)
	Surgical complication	8(29.6%)	Stage of pouch surgery	2	13(48.1%)
	Anismus	1(3.7%)		3	8(29.6%)
Anastomosis	Handsewn	4(14.8%)		redo pouch	6(22.2%)
	Stapled	15(55.6%)	EIM	No	16(59.3%)
	Unknown	8(29.6%)		Yes	11(40.7%)
Gender	Female	14(51.9%)	Autoimmune disease	No	25(92.6%)
	Male	13(48.1%)		Yes	2(7.4%)
Race	Caucasian	23(85.2%)	PSC	No	26(96.3%)
	Others	4(14.8%)		Yes	1(3.7%)
Smoking	No	21(77.8%)	Refractory pouchitis	No	23(85.2%)
	Ex or active	6(22.2%)		Yes	4(14.8%)
Chronic NSAID	No	25(92.6%)	Cuffitis	No	22(81.5%)
use history	Yes	2(7.4%)		Yes	5(18.5%)
Indication of colectomy	Refractory colitis	24(88.9%)	Pouch complication	No	3(11.1%)
	Dysplasia	3(11.1%)		Yes	24(88.9%)
Preoperative diagnosis	UC	25(92.6%)	CD of pouch	No	26(96.3%)
	IC or CD	2(7.4%)		Yes	1(3.7%)

IBD: inflammatory bowel disease; IPS: irritable pouch syndrome; NSAID: non-steroidal anti-inflammatory drugs; UC: ulcerative colitis; IC: indeterminate colitis; CD: Crohn’s disease; EIM: extraintestinal manifestation; PSC: primary sclerosing cholangitis.

**Table 2:** Comparisons of measurements based on pelvic MRI-DIXON-F and CT images.

Measurements	MRI-DIXON-F	CT	P
Age at imaging (y)	41(35–57)	42(36–57)	0.978
Time from pouch construction to imaging (Median and IQR, y)	4(1–10)	4(1–10)	0.986
Weight at imaging (Kg)	69.501 ± 16.698	68.576 ± 16.297	0.838
Height at imaging (m)	1.694 ± 0.113	1.691 ± 0.114	0.899
BMI at imaging (Kg/m^2^)	24.155 ± 5.323	23.890 ± 4.754	0.848
Perianal Fat Thickness (cm)	3.430 ± 0.972	2.862 ± 0.734	<0.001^▴^
	3.230(2.851–3.944)	2.924(2.250–3.337)	<0.001^#^
AreaM (cm^2^)	54.779 ± 22.474	23.831 ± 16.269	<0.001^▴^
	53.171(36.1–76.752)	22.743(8.497–37.331)	<0.001^#^
AreaH (cm^2^)	45.232 ± 20.298	20.553 ± 15.650	<0.001^▴^
	39.941(31.312–56.564)	15.992(6.850–34.258)	<0.001^#^

^▴^Using paired t test; ^#^Using paired Wilcoxon rank sum test. IQR: Interqutile range.

### Validation of measurements of peripouch fat with pelvic MRI-DIXON-F images

**Figure 2:**
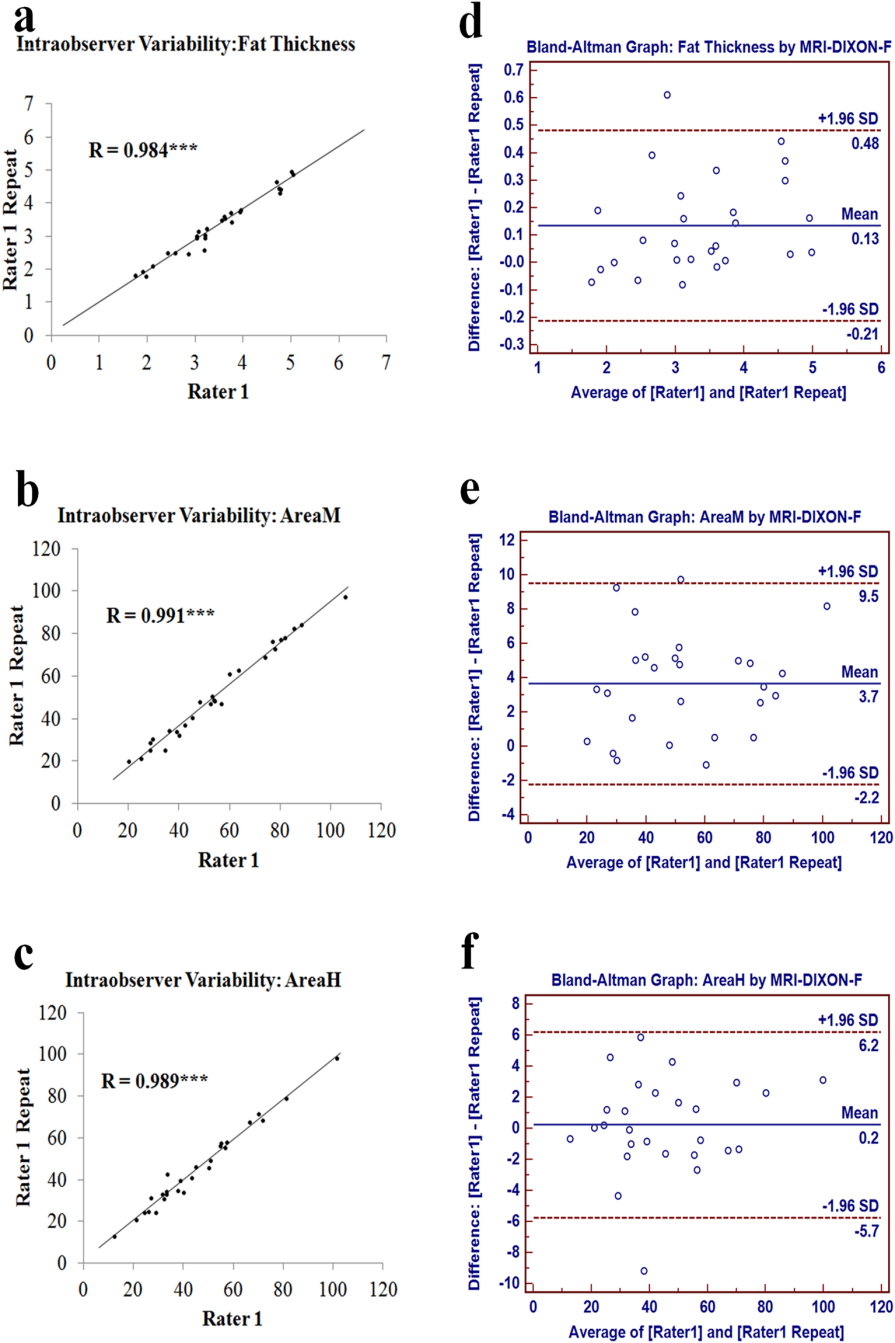
Determination of intraobserver variability for measuring peripouch fat with pelvic MRI-DIXON-F images. Scatter plots of intraobserver discrepancies for perianal fat thickness (**a**), AreaM (**b**) and AreaH (**c**); Bland-Altman graphs (**d**,**e**,**f**) demonstrated variability between two measurements: mean (central blue line) and 95% confidence intervals (upper and lower red lines). ***P < 0.001.

Scatter plots (Fig. 2a,b,c) showed that intraobserver discrepancies were not statistically significant, since two measurements were significantly correlated with each other in perianal fat thickness (correlation efficiency: r = 0.984, P < 0.001), AreaM (r = 0.991, P < 0.001), and AreaH (r = 0.989, P < 0.001). The average difference between two measure, as an estimate of agreement, was all found to be very small, with a difference of 0.135 ± 0.177 cm for perianal fat thickness (limits of agreement: −0.212 to 0.482 cm, (Fig. 2d), 3.650 ± 2.999 cm^2^ for AreaM (limits of agreement: −2.228 to 9.528 cm^2^, (Fig. 2e) and 0.235 ± 3.046 cm^2^ for AreaH (limits of agreement: −5.736 to 6.205 cm^2^, (Fig. 2f). For all three parameters, more than 96.296% (26/27) of dots fell within the limit of agreements (Fig. 2d-f).

**Figure 3:**
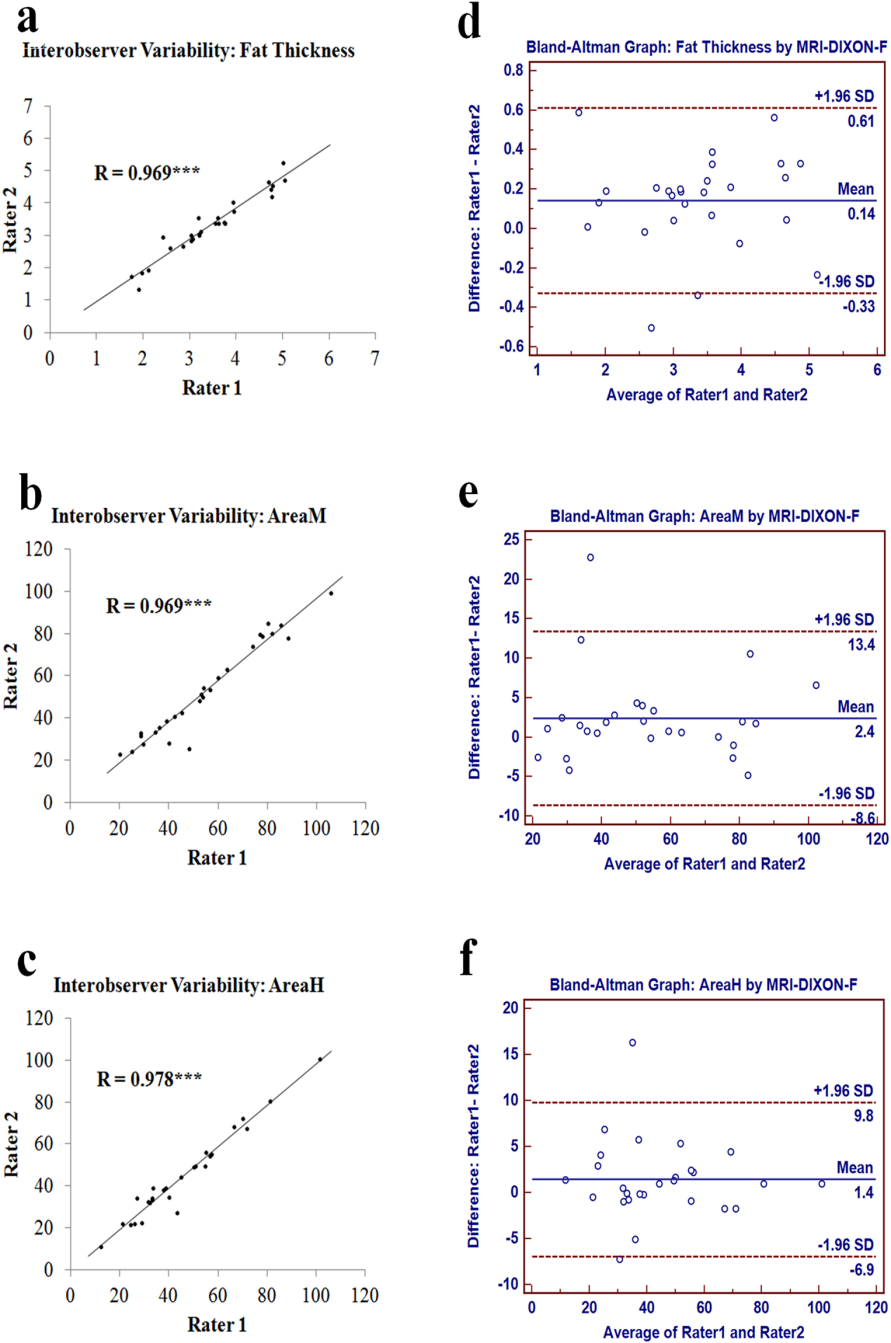
Determination of interobserver variability for measuring peripouch fat with pelvic MRI-DIXON-F images. Scatter plots of interobserver discrepancies for perianal fat thickness (**a**), AreaM (**b**) and AreaH (**c**); Bland-Altman graphs (**d**,**e**,**f**) demonstrated variability in measurements of two raters: mean (central blue line) and 95% confidence intervals (upper and lower red lines). ***P < 0.001.

As far as interobserver discrepancies were concerned, scatter plots identified no significant differences in measurements between two groups in perianal fat thickness (r = 0.969, P < 0.001, (Fig. 3a), AreaM (r = 0.969, P < 0.001, (Fig. 3b), and AreaH (r = 0.971, P < 0.001, (Fig. 3c). The average differences between the two raters’ measurements were very small, with a difference of 0.142 ± 0.240 cm for perianal fat thickness (limits of agreement: −0.328 to 0.611 cm,Fig. 3d), 2.387 ± 5.607 cm^2^ for AreaM (limits of agreement: −8.602 to 13.376 cm^2^, Fig. 3e) and 1.441 ± 4.261 cm^2^ for AreaH (limits of agreement: −6.911 to 9.793 cm^2^, Fig. 3f). For all three parameters, more than 92.593% (25/27) of dots fell within the limit of agreements (Fig. 3d–f).

### Correlation between measurements of pelvic MRI-DIXON-F and CT images

**Figure 4:**
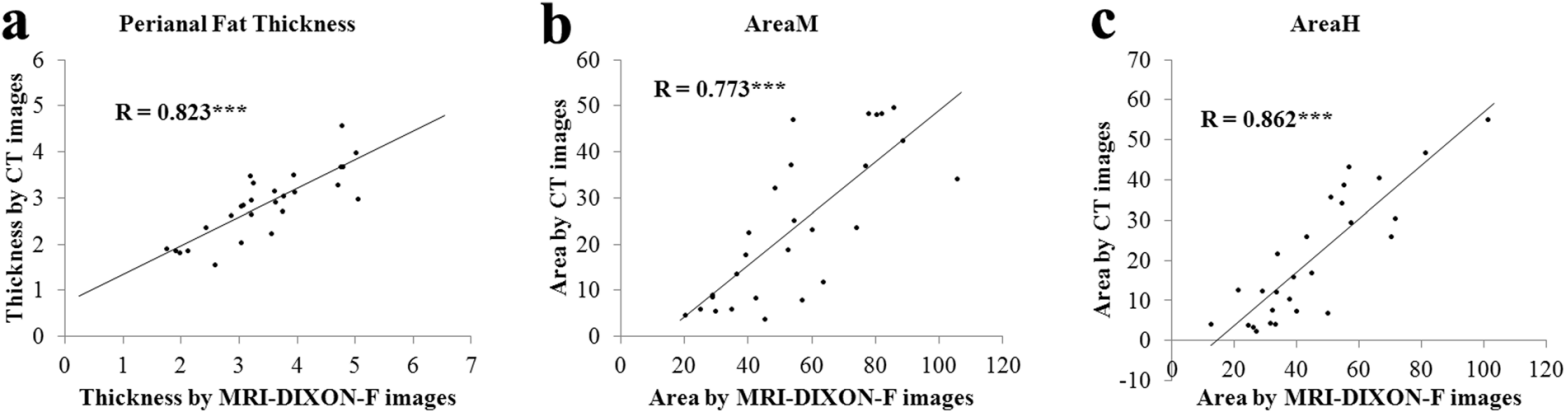
Correlation analysis of measurements obtained from pelvic MRI-DIXON-F and CT images in Perianal Fat Thickness (**a**), AreaM (**b**) and AreaH (**c**). ***P < 0.001.

The measurements of MRI-DIXON-F images were significantly higher than CT images, in terms of perianal fat thickness, AreaM and AreaH (Table 1). However, scatter plots demonstrated that there were highly significant correlations between measurements obtained from CT and MRI-DIXON-F images, in terms of peripouch fat thickness (r = 0.823, P < 0.001,Fig. 4a), AreaM (r = 0.773, P < 0.001, Fig. 4b) and AreaH (r = 0.862, P < 0.001, Fig. 4c). To achieve normalization between two different imaging modalities, regression analyses were also performed to get regression formulas. The formula facilitated the mutual conversion of measurements obtained from pelvic MRI-DIXON-F images and pelvic CT images. Therefore, the equivocal CT/MRI measurements could be calculated using the following formula: Thickness_CT = 0.610 × Thickness_MRI + 0.853; AreaM_CT = 0.865 × AreaM_MRI + 1.392; AreaH_CT = 0.508 × AreaH_MRI + 15.001, when the MRI/CT measurements were known.

## Discussion

We analyzed a total of 27 patients who had both pelvic MRI-DIXON-F and CT images within one year. The median time interval and interquartile range (IQR) between the pelvic CT scan and pelvic MRI-DIXON-F scan was 20 (−90 to 70) days. For pelvic MRI-DIXON-F image, scatter plots and Bland-Altman graph showed that both intraobserver and interobserver discrepancies were extremely small in measuring perianal fat thickness, AreaM and AreaH, and more than 92.593% (25/27) of dots fell within the limit of agreements. In addition, our study demonstrated that measurements based on MRI-DIXON-F images were significantly higher than CT images, in terms of perianal fat thickness, AreaM and AreaH. Nonetheless, there were significantly correlations between measurements obtained from MRI-DIXON-F and CT images. Finally, mathematical formulas for mutual transition between measurements of MRI-DIXON-F and CT images were constructed.

Measurements of abdominal visceral fat using MRI and CT scans were first compared by Seidell in a prospective study including 7 healthy male volunteers^25^. Strong correlations had been reported between MRI and CT images when measuring abdominal visceral fat^25^. Thereafter, several studies had verified the reliability of prospective and retrospective measurements of abdominal visceral fat using CT and MRI^18–21^. The reported results in abdominal visceral fat were consistent with what we found in peripouch fat. In our study, measurements based on MRI-DIXON were significantly higher than CT images, in terms of perianal fat thickness, AreaM and AreaH. These results were contrary to Waduud’s study, which found that MRI measurements of total abdominal area and abdominal waist circumference were lower than the counterpart CT measurements^21^. But in Waduud’s study, CT scan was performed in the supine position while MRI scan was performed in the prone position, and the differences were attributed to different patient positions. But, in our study, both CT and MRI scans were performed in the supine position, so patient position was not a major cause. Potretzke^28^ reported that using CT image to recognize fat could lead to overestimation of abdominal fat due to the incorrect inclusion of colonic content. In our opinions, there are two possible reasons for the higher measurements in MRI-DIXON. Firstly, the MRI-DIXON images are more efficient in depicting peripouch fat than CT images. MRI-DIXON is specific in displaying fat tissue, which is superior to MRI-T1 in depicting water-fat borders and image details^29,30^. In CT scan, only Hounsfield units are used to differentiate fat from adjacent tissue. In the case of IPAA, pouch body is located at the narrow pelvic cavity, and some fat tissue can’t be shown on CT image due to its partial volume effect. Secondly, MRI images might have more motion artefact. Compared with CT scan, MRI scan takes a longer acquisition time, usually greater than a breath-hold, which may lead to more motion artefact. In this study, the fat tissue was differentiated from adjacent non-fat tissue by setting automatic threshold when analyzing MRI-DIXON images. Using this technique, only manual circling of peripouch fat (regions of interest) was required. This approach has the advantage of minimizing interobserver and intraobserver differences, and it is much more subjective and time-saving compared with individualized threshold^21^. We only used a single slice technique rather than a volume-based technique for assessment of peripouch fat. Borkan’s study confirmed that there was no additional advantage of volumetric analysis compared with using multiple levels images in measuring abdominal fat^31^. We initially planned to analyze the peripouch fat area at three levels: the lowest level of pouch, the middle level of the pouch body, and the highest level of pouch. Since the pouch is very close to the ani elevator at the lowest level of pouch, the area of peripouch fat is too small to be analyzed. Therefore, we elected to measure the perianal fat thickness.

In our Pouch Center, we have routinely used pelvic MRI to assess fistula, abscess and anastomotic leak of the pouch, with its higher resolution than CT. However, CT imaging has the advantage of being quicker to acquire, which avoids potential interference of bowel movement on region of interest measures^18,24,32^. CT can also be used in patients when MRI is contraindicated, for instance, patients with intrauterine device or other implanted metallic objects in pelvic cavity. In addition, CT is more cost effective than MRI, leading to its more frequent use in clinical practice. However, CT scan is associated with a significant dose of radiation, which restricts its application in certain patients, such as pregnant women. MRI does not involve radiation exposure, which allows for frequent repeat imaging and it could be applied in at-risk patients, such as pregnant women^18^. Furthermore, MRI is superior to CT in measuring fat, as fat has a typical short longitudinal relaxation time which makes it easy to be differentiated from other adjacent tissues in MRI image^21^. Since both CT and MRI scans have their own advantage, disadvantage, indication and contraindication, respectively, so it is very important to explore whether they are interchangeable. The MRI-DIXON is a T1 weighted gradient echo sequence, which displays fat and water separately^29,30,33^. MRI-DIXON has four images (in phase, opposed phase, water phase and fat phase) which could be gained in a single scan, and the fat phase (shorted as MRI-DIXON-F) is specialized in exhibiting fat tissue^29^. MRI-DIXON-F image is superior to standard MRI-T1 image in depicting water-fat borders and image details^30^, and it is reported to be associated with a high interobserver reliability in measuring fat area around biceps^33^.

This is the first study to compare measurements of peripouch fat using retrospective CT and MRI-DIXON images. The CT and MRI-DIXON scans used in this study were all performed without pre-defined research criteria, so it exactly represented current clinical practice. We demonstrated that the intraobserver and interobserver variability for measurements based on MRI images were extremely small in all three parameters. It validated that MRI image was a robust and reproducible method for measuring peripouch fat. Strong positive linear correlations had been identified and mathematical formula had been developed to normalize measurements between imaging modalities.

The measurements were easily gotten from retrospective CT and MRI images, so it provided an opportunity for researchers and clinicians to determine peripouch fat change without any additional cost. Our clinical practice and primary research results of another study indicated that, the peripouch fat area had something to do with chronic pouchitis and pouch failure. So the dynamic monitoring of peripouch fat might be helpful in the prediction of chronic pouchitis and pouch failure. Since both CT imaging and MRI imaging have their own advantages, so they are mutually supplemental in the routine follow up and complication evaluation in IBD patients. The conclusion of this study would be practically used to facilitate depicting the dynamic alteration of peripouch fat, and researching on the underlying mechanism between peripouch fat and pouch complication.

There were some limitations in this study. Firstly, the small sample size was a major limitation. The 1,863 pouch patients in our database were not followed up routinely by pelvic CT or MRI scan. For most patients, CT or MRI scan was enforced to evaluate pouch complications, such as anastomatic stricture, perianal abscess and pouch fistula. Furthermore, MRI-DIXON was not routine series for pelvic MRI scan, and only approximate 50% of pelvic MRI scans had MRI-DIXON series in our clinic. Therefore, selection bias was inevitable. Secondly, timing gap between CT and MRI scan was also a potential confounding factor. The CT and MRI images included in our analyses were taken at different time points. During these periods, weight loss or gain may happen, which might interfere with the reliability of comparison analysis. Waduud’s study verified that the timing of imaging did not affect the linear relationship of measurements on CT and MRI^21^. Since one year was only a relatively short period as compared with the long survival period of pouch, and our study still showed that there were strong positive correlations between measurements of CT and MRI. This suggested CT and MRI images performed up to a year apart could be reliably analyzed and compared as demonstrated in our study. Thirdly, some MRI images were incomplete and didn’t include whole area of pelvic, so it was impossible for us to calculate subcutaneous fat area. Instead, perianal fat thickness was applied. Finally, most pelvic CT or MRI images didn’t reach the height of umbilical level or the third lumbar vertebrae level, which was often used in measuring the abdominal fat distribution. Therefore, the total abdominal fat distribution and total abdominal visceral fat were not analyzed in this study. Despite the above mentioned limitations, strong relationships were still demonstrated between MRI-DIXON-F and CT measurements in this study.

In conclusion, our study demonstrates that measurements of peripouch fat based on pelvic MRI-DIXON-F image is feasible and highly reproducible for pouch patients with underlying IBD, since both intraobserver and interobserver differences are extremely small. Pelvic MRI images and CT images are interchangeable in retrospective measurements of peripouch fat, which will provide new revenue to the prospective as well retrospective assessment of fat and mesentery in other gastrointestinal disorders.

## Methods and Materials

### Patients

This study was performed using pelvic MRI-DIXON-F and CT images from our Center for Ileal Pouch Disorders. Patients were identified from a prospectively maintained Institutional Review Board approved Pouch Database between 2002 and 2016 in Cleveland Clinic. Demographics, comorbidities, pouch complications and pouch outcomes were all prospectively maintained in the pouch database.

### Inclusion and Exclusion Criteria

The inclusion criteria were patients who had (1) underlying UC, Crohn disease (CD) and indeterminate colitis; (2) one or more pelvic MRI-DIXON scans in our electronic medical images systems between pouch construction date and pouch failure date; (3) one or more pelvic CT scans in our electronic medical systems between pouch construction inception date and pouch failure date; and (4) the interval between CT scan and MRI-DIXON scan was less than one year.

Exclusion criteria were patients with (1) pelvic MRI images but no DIXON series; (2) radiation or chemotherapy between CT and MRI; and (3) underlying familial adenomatous polyposis.

### Image analysis and data collection

**Figure 5:**
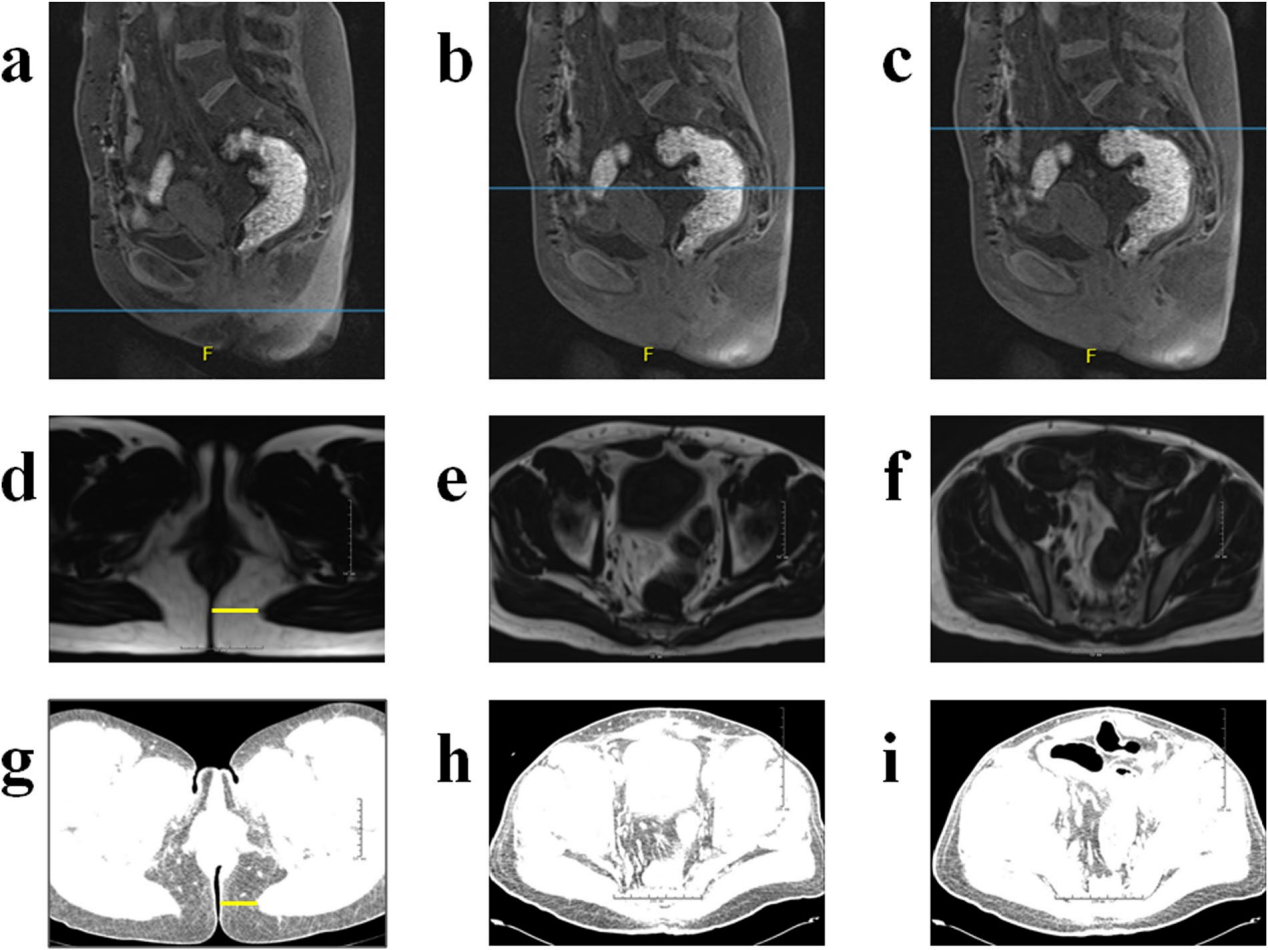
Sagittal MRI-DIXON-F images at the anal verge level (Level 1, (**a**)), the middle height level of pouch (Level 2, (**b**)) and the highest level of pouch (Level 3, (**c**)). Transverse cross-section MRI-DIXON-F images at Level 1 (**d**), Level 2 (**e**), Level 3 (**f**). Transverse cross-section CT images of the same patient at Level 1 (**g**), Level 2 (**h**), Level 3 (**i**).

Both pelvic MRI-DIXON-F and CT images were analyzed for all patients. Transverse cross-sectional images were obtained in the guide of sagittal image at the level of (1) the anal verge level (level of levator ani origin, Level 1, Fig. 5a); (2) the middle height level of pouch (Level 2, Fig. 5b); (3) the highest level of pouch (Level 3, Fig. 5c). CT images were reset using the CT Hu (−30 to −190) in workstation^18,34^. Both the pelvic MRI-DIXON-F images (Fig. 5d–f) and CT images (Fig. 5g–i) with rulers were downloaded from the digital imaging system at the above mentioned 3 levels, and stored in a specific file using the patients’ medical record number. Each MRI image was analyzed by two individual raters to identify interobserver difference. Both raters, at the time of measuring, were blinded to clinical data and the measurements of the other rater. Images were analyzed for two times by the same rater to identify intraobserver differences.

**Figure 6:**
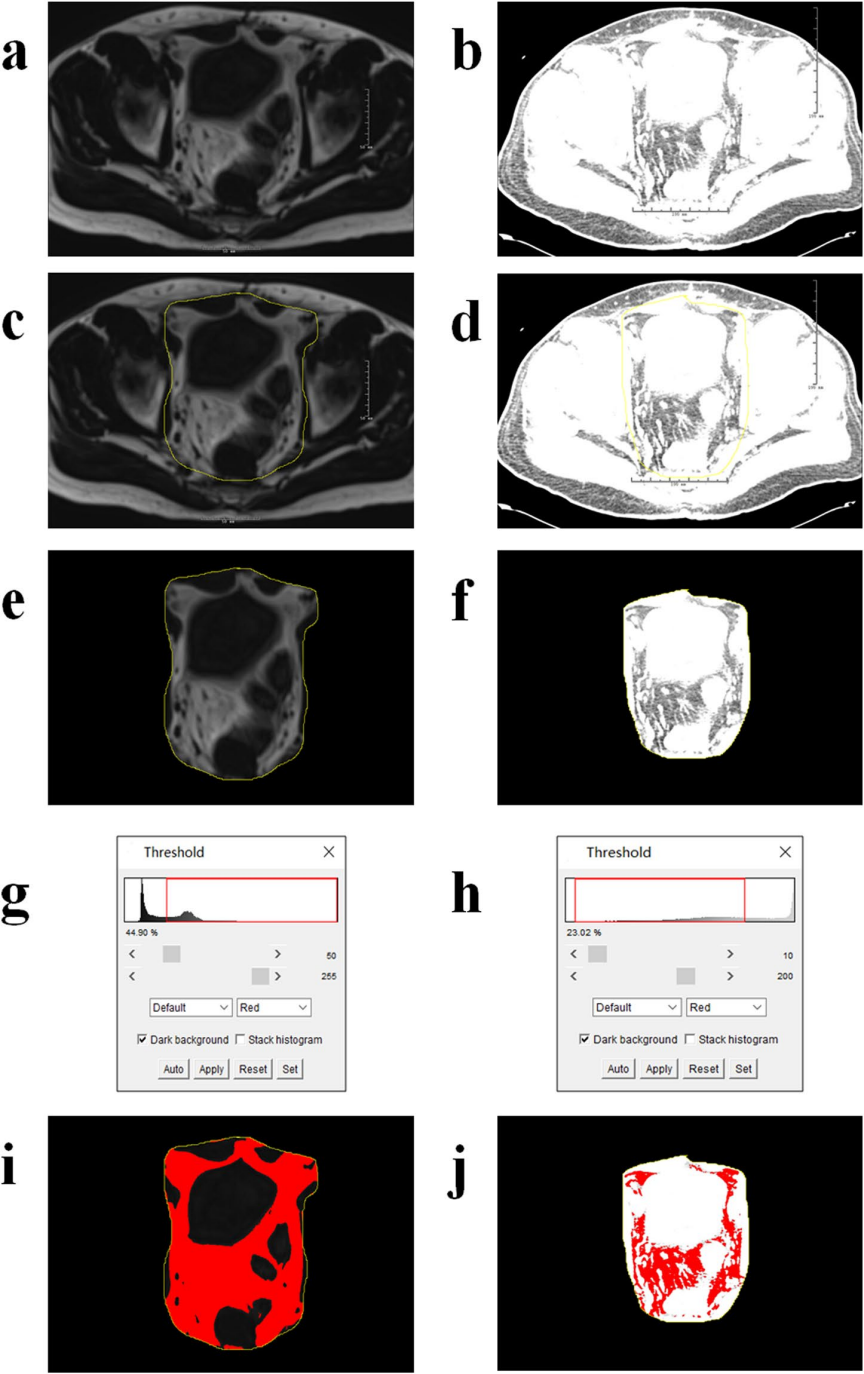
Process of measuring AreaM using both pelvic MRI-DXION-F images (**a**,**c**,**e**,**g**,**i**) and pelvic CT images (**b**,**d**,**f**,**h**,**j**) in the same patients. Circle peripouch fat (**c**,**d**), clear outside irrelevant regions (**e**,**f**), set thresholds (**g**,**h**), and finally calculate peripouch fat area (**i**,**j**).

The thickness of perianal subcutaneous fat was calculated at Level-1 (Fig. 5d,g) after setting scales in the guide of the attached ruler. The peripouch fat areas were calculated at Level-2 (middle peripouch fat area, AreaM) and Level-3 (highest peripouch fat area, AreaH). Fat thickness and fat areas were calculated using the ImageJ software (http://imagej.nih.gov/ij/). For all patients, perianal fat thickness, AreaM and AreaH were measured in both pelvic MRI-DXION-F and CT images. Figure 6 showed the process of measuring AreaM using both MRI-DXION-F and CT images (Fig. 6a,b). Firstly, the peripouch fat (regions of interest) was circled manually (Fig. 6c,d). Briefly, only the pelvic visceral fat, and the pelvic parietal fat inside the pelvic muscle and the abdominal wall muscle were included. The subcutaneous fat and the fat in the bone marrow of the sacrum, femoral head and lateral pelvic wall, were excluded. And these muscles act as inherent boundary of the peripouch fat area. And then the outside redundant tissue was cleared (Fig. 6e,f). Images were transformed into 8-bit type, and then the thresholds were adjusted. For MRI-DXION-F images, the threshold was automatically set at the midpoint between the two peaks of the identified signal intensities in the histograms (Fig. 6g), and fat area was highlighted automatically in almost all images^21^. For CT images, the thresholds were set at a fixed range (10–200) (Fig. 6h). The red area demonstrated peripouch fat (Fig. 6i,j). And then the red areas were calculated with the “Analyze Particles” tool in the ImageJ software.

### Validation of measurements based on MRI images

In order to determine the reliability of the measurement method, intraobserver and interobserver variability was assessed Intraobserver variability was evaluated by comparing repeated measurements made by a single rater. Interobserver variability was evaluated by comparing the measurements made by two independent raters. A formative assessment of intraobserver and interobserver variability was performed by scatter plot with linear regression and Bland-Altman graph.

### Statistical analysis

A paired t test or Wilcoxon rank sum test was used for continuous variables as appropriate. Fisher exact or Chi-square test was used for categorical variables as appropriate. Interobserver and intraobserver differences were evaluated by scatter plot with linear regression and Bland-Altman plots. For the Bland-Altman plots, the limits of agreement were calculated from the difference between the raters’ measurements for thickness, AreaM and AreaH, respectively. P < 0.05 (two-side) was considered as statistically significant. Bland-Altman graphs were drawn using the MedCalc 9.2.0. All other analyses were carried out using the SPSS 17.0 (Chicago, IL).

### Compliance with ethical standards

The study was approved by the Institutional Review Board of Cleveland Clinic. For this type of study formal consent is not required.
